# Aqueous Extracts of *Rhus trilobata* Inhibit the Lipopolysaccharide-Induced Inflammatory Response In Vitro and In Vivo

**DOI:** 10.3390/plants13202840

**Published:** 2024-10-10

**Authors:** Alejandra Jazmín Rodríguez-Castillo, Susana Aideé González-Chávez, Ismael Portillo-Pantoja, Eunice Cruz-Hermosillo, César Pacheco-Tena, David Chávez-Flores, Ma. Carmen E. Delgado-Gardea, Rocío Infante-Ramírez, José Juan Ordaz-Ortiz, Blanca Sánchez-Ramírez

**Affiliations:** 1 Programa de Doctorado en Ciencias Químicas, Facultad de Ciencias Químicas, Universidad Autónoma de Chihuahua, Circuito Universitario Campus II, Chihuahua 31125, Mexico; p286463@uach.mx (A.J.R.-C.); ismanq2717@gmail.com (I.P.-P.); eunice.crh@gmail.com (E.C.-H.); dchavezf@uach.mx (D.C.-F.); mcdelgado@uach.mx (M.C.E.D.-G.); rinfante@uach.mx (R.I.-R.); 2 Laboratorio PABIOM, Facultad de Medicina y Ciencias Biomédicas, Universidad Autónoma de Chihuahua, Circuito Universitario Campus II, Chihuahua 31125, Mexico; sagonzalez@uach.mx (S.A.G.-C.); dr.cesarpacheco@gmail.com (C.P.-T.); 3 Laboratorio de Metabolómica y Espectrometría de Masas, Unidad de Genómica Avanzada, CINVESTAV-IPN, Km. 9.6 Libramiento Norte Carr. Irapuato-León, Irapuato 36824, Mexico; jose.ordaz.ortiz@cinvestav.mx

**Keywords:** cyclooxygenase, inflammation, interleukin-1, macrophages, paw edema, prostaglandin E2, *Rhus trilobata*

## Abstract

Chronic noncommunicable diseases (NCDs) are responsible for approximately 74% of deaths globally. Medicinal plants have traditionally been used to treat NCDs, including diabetes, cancer, and rheumatic diseases, and are a source of anti-inflammatory compounds. This study aimed to evaluate the anti-inflammatory effects of *Rhus trilobata* (Rt) extracts and fractions in lipopolysaccharide (LPS)-induced inflammation models in vitro and in vivo. The aqueous extract (RtAE) and five fractions (F2 to F6) were obtained via C18 solid-phase separation and tested in murine LPS-induced J774.1 macrophages. Key inflammatory markers, such as IL-1β, IL-6, TNF-α, and COX-2 gene expression were measured using RT-qPCR, and PGE_2_ production was assessed via HPLC-DAD. The in vivo effects were tested in an LPS-induced paw edema model in Wistar rats. Results showed that RtAE at 15 μg/mL significantly decreased IL-1β and IL-6 gene expression in vitro. Fraction F6 further reduced IL-1β, TNF-α, and IL-6 gene expression, COX-2 expression, and PGE_2_ production. In vivo, F6 significantly reduced LPS-induced paw edema, inflammatory infiltration, and IL-1β and COX-2 protein expression. Chemical characterization of F6 by UPLC/MS-QTOF revealed at least eight compounds with anti-inflammatory activity. These findings support the anti-inflammatory potential of RtAE and F6, reinforcing the medicinal use of Rt.

## 1. Introduction

Chronic noncommunicable diseases (NCDs), such as systemic lupus erythematosus, multiple sclerosis, and rheumatoid arthritis, significantly impact the healthcare system [1]. It is estimated that 74% of deaths globally are caused by NCDs [2]. In developing countries such as Mexico, public health systems face many challenges in caring for these diseases efficiently, and most patients have difficulty accessing private care due to high costs. All of these diseases commonly involve the development of an ongoing inflammatory process [3].

Inflammation is a complex set of interactions between soluble factors and cells that lead to the healing of damaged tissue; these interactions can be acute or chronic, the latter generating health consequences [4]. During inflammation, activated macrophages (MOs) produce chemical mediators, such as prostaglandin E2 (PGE_2,_) through the induction of the cyclooxygenase (COX)-2 isoenzyme [5], as well as pro-inflammatory cytokines such as interleukin (IL)-1β, IL-6, and tumor necrosis factor (TNF)-α [6,7]. IL-1β acts in the innate immune response on several cell types, mainly on MOs [8,9]. IL-6 has several functions, primarily promoting cell growth and differentiation and mediating the acute phase response to the onset of inflammation [10]. TNF-α is involved in transendothelial differentiation and migration, leukocyte adhesion, and apoptosis [11]. Excessive production of pro-inflammatory cytokines or dysregulation of this process can lead to inflammation, resulting in tissue damage, hemodynamic changes, and organ failure [12].

The discovery of plant secondary metabolites that inhibit the production of pro-inflammatory mediators with a positive effect on the resolution of inflammation is of increasing interest to the scientific community [13,14].

*Rhus trilobata* Nutt. (Rt; *Anacardiaceae*) is an endemic plant of Chihuahua State in Mexico, which grows in oak forests and wetlands and is widely found from southern Canada to central Mexico [15]. *Rhus* species have been used in several applications for stomach disease, diarrhea, sore throat, and arthritis. Rt is traditionally used to treat gastrointestinal diseases and cancer; however, scientific information on its use and potential pharmacological properties are scarce [16]. Recently, our research group reported the antineoplastic effect of the aqueous extract of Rt stems (RtAE) in colon and ovarian cancer cell lines. Additionally, in vivo toxicological studies in mice revealed that intraperitoneal administration of 200 mg/kg RtAE or an active antineoplastic fraction caused significant changes neither in behavioral or histological parameters nor in biochemical markers [17,18]. However, slight leukopenia in mice treated with RtAE and the antineoplastic fraction at 14 days posttreatment was detected, although no studies have been conducted to clarify this point [18]. Chemical characterization of RtAE revealed high contents of polyphenols, such as phenolic acids and flavonoids, which are reportedly antioxidant and anti-inflammatory compounds [17,18].

The components included methyl gallate, epigallocatechin 3-cinnamate, quercetin 3-(2″-galloylglucosyl)-(1→2)-alpha-L-arabinofuranoside, 1,2,3,4,6-pentakis-O-galloyl-β-D-glucose (β-PGG), 4-O-digalloyl-1,2,3,6-tetra-O-β-D-galloylglucose, myricetin 3-(4″-galloylrhamnoside), and fisetin, some of which have been reported to have anti-inflammatory effects [19,20,21]. Some of these compounds have been studied in extracts from other plants of the *Rhus* genus; for example, dyhidrofisetin, a polyphenol compound derived from fisetin present in *Rhus verniciflua* Stokes, significantly reduced TNF-α, IL-1β, IL-6, and monocyte chemotactic protein (MCP-1) in carrageenan-stimulated RAW 264.7 macrophages or in in vivo carrageenan-induced mouse paws [22]. Since inflammation is a multistep cascade, in vitro studies are required to screen for anti-inflammatory activity and discover cell targets; moreover, in vivo studies allow the evaluation of cytokine modulation. In this study, we first evaluated the effect of RtAE and fractions obtained by solid phase separation on the mRNA expression of IL-1β, TNF-α, IL-6, and COX-2 in cultures of macrophages (MOs) J774A.1 stimulated in vitro with lipopolysaccharide (LPS). After that, we evaluated the effect of RtAE and fractions in the LPS-induced paw edema model. LPS-induced paw edema is an acute inflammation in vivo model that helps evaluate TNF-α, IL-1β, and COX-2 expression [23].

## 2. Results

### 2.1. Effect of Rhus trilobata Extracts on MO Cell Viability

**Figure 1:**
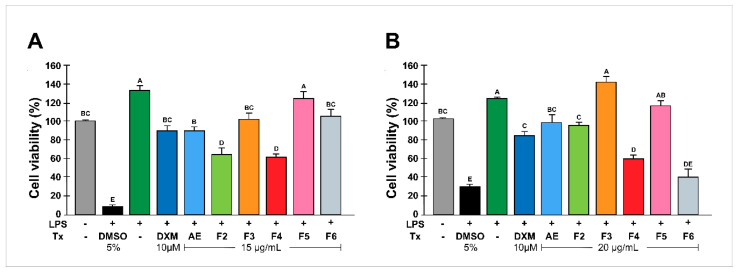
Effect of RtAE and fractions on the viability of LPS-treated MOs. The J774.1 MOs were stimulated for 24 h with 5 μg/mL LPS and treated for 24 h with each RtAE or fraction at 15 µg/mL (**A**) or 20 µg/mL (**B**). The bars show the mean ± SD of three biological replicates (*n* = 3, in triplicate). ANOVA and Tukey’s test (95% confidence) were used to determine group differences. Bars that do not share a letter differ significantly (*p* ≤ 0.05). AE, aqueous extract; DXM, dexamethasone; DMSO, dimethyl sulfoxide; F, fraction; LPS, lipopolysaccharides; Tx, treatment.

To assess whether RtAE and its fractions had cytotoxic effects on J774.1 MOs, cell viability was analyzed by MTT assays at 24 h. As depicted in Figure 1A, at 15 μg/mL RtAE, fractions F3, F5, and F6 did not significantly decrease cell viability compared with that of nonstimulated cells, and F2 and F4 did not reduce viability by more than 40% (Figure 1A). At 20 μg/mL, compared with nonstimulated cells, cells treated with RtEA or F2 significantly decreased MOs viability. In the cultures treated with F4 and F6, cell viability was reduced by more than 60% (Figure 1B). Therefore, 15 μg/mL was used to evaluate the secretion of inflammatory mediators in LPS-induced MOs.

### 2.2. Rhus trilobata Anti-Inflammatory Activity in LPS-Induced MOs

**Figure 2:**
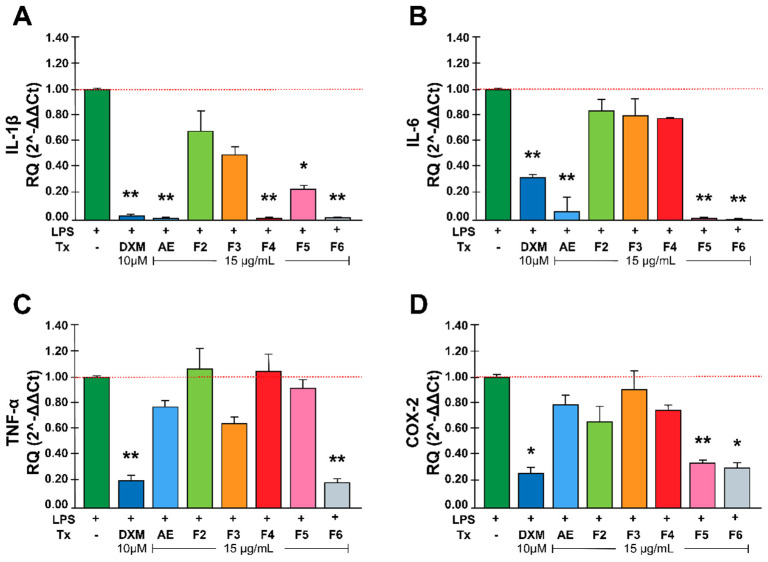
Effect of RtAE and its fractions on inflammatory mediators in LPS-induced MOs. The J774.1 MOs were stimulated for 24 h with 5 μg/mL LPS and treated for 24 h with each RtAE or fraction at 15 µg/mL. The relative RNA expression of IL-1β (**A**), IL-6 (**B**), TNF-α (**C**), and COX-2 (**D**) was evaluated via RT-qPCR using the 2^−ΔΔCt^ method. The bars show the mean ± SD of the relative quantification of cDNA from two independent experiments performed in duplicate. A *t*-test was used to determine differences compared with the non-treated group. * *p* ≤ 0.05; ** *p* ≤ 0.01. LPS, lipopolysaccharide; (−), LPS-induced edema without treatment; DXM, dexamethasone; Tx, treatment; AE, aqueous extract; F, fraction.

To analyze the effect of RtEA and the fractions on the expression of inflammatory mediators, the mRNA expression of IL-6, TNF-α, IL-1β, and COX-2 was quantified via RT-qPCR. Figure 2A shows that treatment with RtEA, F4, F5, or F6 significantly decreased IL-1β expression in LPS-stimulated MOs; RtEA, F4, or F6 had an anti-inflammatory effect of approximately 90%, comparable to that of DXM. A similar effect on IL-6 expression was obtained with RtEA, F5, and F6 (Figure 2B). Interestingly, only F6 treatment significantly decreased TNF-α RNA expression (Figure 2C). Finally, F5 and F6 significantly reduced COX-2 RNA expression (Figure 2B).

**Figure 3:**
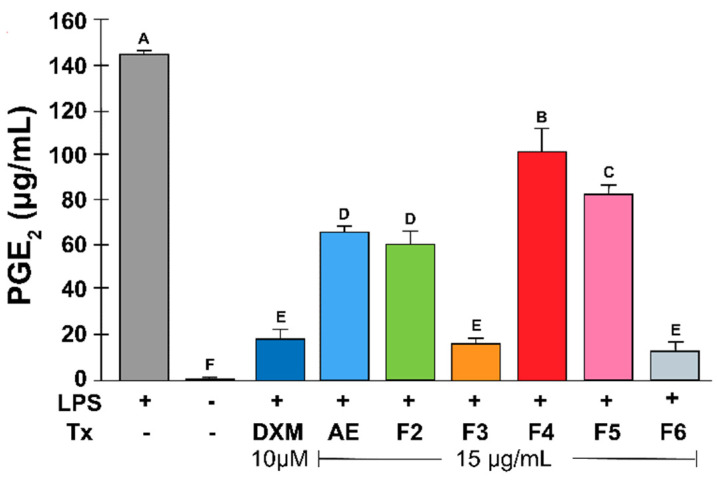
Effect of RtAE and its fractions on PGE_2_ release by LPS-induced MOs. The J774.1 MOs were stimulated for 24 h with 5 μg/mL LPS and treated for 24 h with each RtAE or the fractions at 15 µg/mL. PGE_2_ released in culture supernatants was quantified via HPLC using a PGE_2_ standard curve. The bars show the mean ± SD of the PGE_2_ quantification of three independent experiments. ANOVA and Tukey’s test (95% confidence) were used to determine group differences; bars that do not share a letter differ significantly (*p* ≤ 0.05). LPS, lipopolysaccharides; (−), LPS-stimulated without treatment; DXM, dexamethasone; Tx, treatment; AE, aqueous extract; F, fraction.

Table 1 shows the percentage of decreased PGE_2_ production by RtAE and fractions. Compared with LPS-stimulated MOs, control MOs not stimulated or treated with DXM showed no significant increase in PGE_2_ production (Table 1 and Figure 3). DXM was the most effective treatment, significantly decreasing PGE_2_ production compared with control of LPS-stimulated MOs, followed by the F6 and F3 treatments; these fractions showed no statistical difference with DXM control. Fractions F2 and RtAE decreased PGE_2_ release by half, followed by F5 and F4, which were the least effective.

### 2.3. In Vivo Evaluation of the Anti-Inflammatory Effects of RtAE and Its Fractions

The anti-inflammatory effects of RtAE and its fractions were evaluated in an LPS-induced paw edema model. For RtAE and fractions, the concentration of 500 µg was established since, in previous work, a single dose of 200 mg/kg body weight was found to be nontoxic [18]. After 12 h of stimulation, all the rats exhibited significantly greater right paw thickness (*p* ≤ 0.05) than on day 0. As shown in Table 2, treatment with 500 µg of DXM significantly reduced inflammation by 93% (*p* ≤ 0.005). Treatment with 500 µg of RtAE, F3, or F6 had a significant anti-inflammatory effect, confirming the findings of the in vitro experiments. Treatment with F2 or F4 did not significantly reduce inflammation; therefore, these fractions were excluded from the subsequent tests. Hence, the anti-inflammatory effects of AE, F3, and F6 at 750 and 1000 µg were evaluated (Table 3).

Increasing the dose to 750 µg increased the anti-inflammatory effects of RtAE (Table 3). However, a dose-dependent effect was not observed since an increase in the concentration of 1000 µg decreased the inhibition. For F3 and F6, increasing concentration decreased the anti-inflammatory effects. Therefore, the most significant reduction of inflammation was achieved with RtAE (80% at 750 µg), followed by F6 (64% at 750 µg) and F3 (59% at 500 µg). Thus, histopathological changes and COX-2 and IL-1β protein expression were analyzed in these groups by IHC.

**Figure 4:**
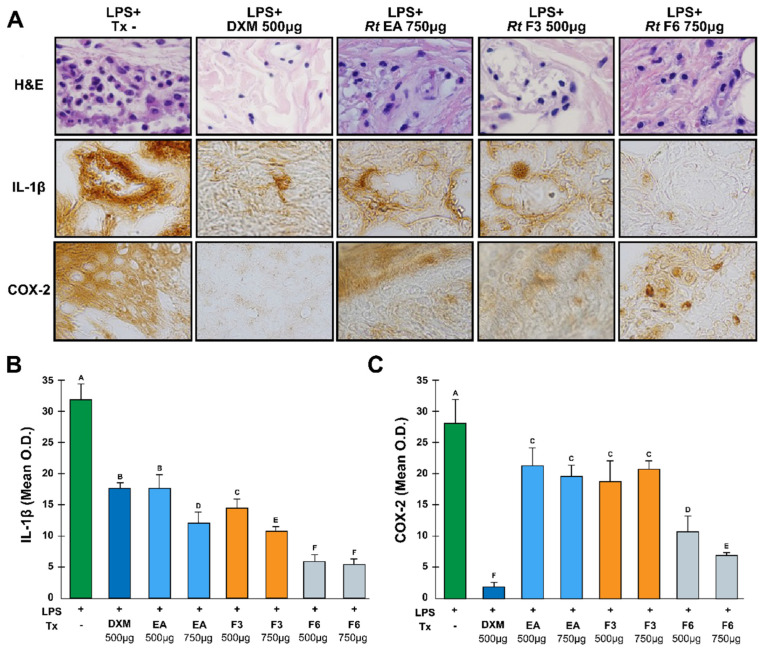
Anti-inflammatory effects of the RtAE, F3, and F6 fractions on IL-1β and COX-2 expression. An LPS-induced paw edema model was generated in Wistar rats, and treatments with RtAE, F3, or F6 were evaluated. The morphological alterations of the footpads were evaluated by H&E staining ((**A**), line 1). The expression of IL-1β ((**A**), line 2) and COX-2 ((**A**), line 3) was evaluated via IHC. The means and SDs of the O.D.s were determined for each study group and are shown for IL-1β (**B**) and COX-2 (**C**). Rats with LPS-induced paw edema treated with 500 μg DXM or left untreated were included as anti-inflammatory positive and negative controls, respectively. ANOVA and Tukey’s test (95% confidence) were used to determine group differences. Bars that do not share a letter differ significantly (*p* ≤ 0.05). LPS, lipopolysaccharides; (−), LPS-induced edema without treatment; DXM, dexamethasone; Tx, treatment; AE, aqueous extract; F, fraction.

Histopathological analysis of the LPS-induced edema controls revealed that the cellular infiltrate was composed mainly of macrophages and neutrophils in the connective tissue and the *stratum spinosum*. A decrease in cellular infiltration was evident in tissues treated with DXM (control), followed by F3 and F6 (Figure 4A).

The signals for IL-1β and COX-2 in the samples were detected in cells from connective tissue (Figure 4A). The quantification of protein expression by O.D. measurements revealed high levels of both proteins in the tissues of LPS-stimulated and untreated footpads (Figure 4B,C). Treatment with DXM significantly inhibited the expression of IL-1β and COX-2 by 45% and 90%, respectively (Figure 4B,C). Treatment with 750 µg of RtAE suppressed, to a greater degree, IL-1β expression by 62%; F3 also decreased IL-1β expression (67% with 1000 µg), and F6 had the most significant inhibitory effect on 83% of the proteins (Figure 4B). No significant difference was found among the different concentrations of RtAE and F3, which induced a significant decrease in COX-2 protein expression from 28 to 41%, respectively (Figure 4C). Conversely, for F6, an increase in dose had a favorable effect only on COX-2 expression.

### 2.4. Phytochemical Composition of F6

**Figure 5:**
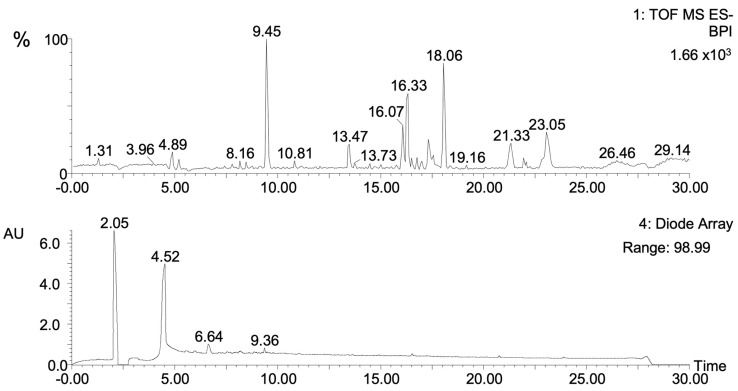
Chromatographic profile of RtAE F6. The F6 fraction of RtAE was analyzed (**A**) by UPLC/MS-QTOF; and (**B**) UPLC-PAD in a wavelength range from 190 to 680 nm. The main compounds are indicated by their corresponding order of appearance. The putative compounds detected in F6 are listed in Table 4 (also see Supplementary Materials).

The metabolite profile of F6 included at least 23 compounds, as revealed by UPLC/MS-QTOF analysis (Figure 5). The putative compounds were identified by their fragmentation patterns and database search (Supplementary material). A search for possible activity was performed (Table 4), resulting in three compounds with anti-inflammatory activity reported previously in another plant genus: Butanamide (**5**), Tulipalin B (**10**), and Genipic acid (**12**). Other compounds detected in F6 with anti-inflammatory activity but nonreported in plants were also detected (**6**, **8**, **11**, **15**, and **16**). Compounds **1**, **7**, **18**, **20**, **21**, and **22** have not been reported in plants and have no biological activity related. Other biological activity-related molecules were antiviral (**2**), antioxidant, and neuroprotective (**3**, **10**, **17**, and **22**). Only one compound was not identified in the database (**19**). None of these compounds have been reported for the *Rhus* genus or specifically in *Rhus trilobata*.

## 3. Discussion

The results presented here demonstrate that RtAE contains compounds that regulate inflammatory mediators and cytokine release in both in vivo and in vitro inflammatory models.

First, in in vitro experiments, the concentration used for assay did not compromise the integrity of the J744.1 MOs since the cytotoxic effect of the RtAE or their fractions did not decrease more than 60% cell viability supporting the observed effect on PGE_2_ and cytokine production.

One of the main objectives in discovering new therapies for treating acute or chronic inflammatory disorders is the inhibition of inflammatory mediators [42]. Second, we quantified the mRNA expression of pro-inflammatory mediators and enzymes (IL-1β, IL-6, TNF-α, and COX-2) produced by LPS-stimulated macrophages [43]. The results showed that RtAE, F4, F5, and F6 significantly decreased IL-1β expression. 

Varela-Rodríguez et al. (2019) identified several compounds with anti-inflammatory activity in RtAE [18]. Based on column retention times, RtAE contains active compounds, such as dihydrofisetin [22], quercetin [44], fisetin [21], butein [45,46], amentoflavone [47], and stigmastane [48]. These compounds have been previously reported to exhibit inhibitory effects on cytokine production, which may explain the results obtained with the RtAE.

For IL-6, AE, F5, and F6 decreased the expression of this cytokine (*p* ≤ 0.001, *p* ≤ 0.05). Previous studies have shown that extracts of several *Rhus* genus plants reduce IL-6 mRNA expression through the action of compounds such as diospyrin, fisetin, butein, and dihydrofisetin [21,22,49,50].

In addition, COX-2 mRNA expression was inhibited by F5 and F6, which could be mediated by IL-1β inhibition. In addition, F6 significantly inhibited PGE_2_ synthesis, which correlated with decreased COX-2 mRNA expression. Additionally, the inhibition of PGE_2_ production by F3 could be related to a specific effect on enzyme or protein synthesis. The compounds included in F3 are polyphenols such as gallic acid and ethyl gallates; studies performed in *Rhus verniciflua* have reported that some compounds, such as butein and dihydrofisetin, can suppress PGE_2_ production in RAW 264.7 macrophages and primary human osteoarthritis chondrocytes, respectively [22,51]. 

Interestingly, TNF-α expression was suppressed only by F6. Studies performed in other *Rhus* species, such as *Rhus verniciflua* stokes, *Rhus coriaria* L., and *Rhus succedanea* L., have demonstrated that their extracts significantly inhibited TNF-α expression and production. Some of the compounds isolated from these plants include butein, 1, 2, 3, 4, 6, penta-O-galloyl beta D-glucose, fisetin, and dihydrofisetin [21,22,45] methyl gallate, epigallocatechin 3-cinnamate, quercetin 3-(2″-galloylglucosyl)-(1→2)-alpha-L-arabinofuranoside, β-PGG, 4-O-digalloyl-1,2,3,6-tetra-O-β-D-galloylglucose, fisetin, and dihydrofisetin [21,22,52], which are also RtAE components, as Varela-Rodríguez et al. reported [17]. 

Given F6′s capacity to suppress TNF-α and other cytokines, it was characterized using UPLC/MS-QTOF. The metabolites identified in this fraction include compounds previously reported for their anti-inflammatory properties, such as 3-(acetamidomethylene)-2-(hydroxymethyl)succinate [35,36,37], 2-(methylthio)-3H-phenoxazin-3-one [33], Butanamide [27], 2-Fluoro-araat [53,54], Tulipalin B [31,32], and Genipic acid [34,55]. Several of these compounds have also been isolated from plants other than Rt. This is the first report to our knowledge of the presence of these compounds in a plant of the *Rhus* genus, specifically in *Rhus trilobata*.

In addition, 3-(acetamidomethylene)-2-(hydroxymethyl)succinate(2-) [56], Adenophostin A [29,57], 2-Fluoro-araATP [53,54], ADP-L-glycero-D-manno-heptose(2-) [26], N-undecanoylglycine [26,58], and Tulipalin B [31,59] showing promising results in modulating immune responses.

Additionally, several other compounds whose biological effects have not been previously reported were identified during the characterization of the F6 fraction. Further research is required to explore the biological roles of these newly identified compounds, both those with biological activity and those yet to be studied. This investigation is essential to determine which specific compounds are responsible for the observed effects or to identify the metabolic and immunological pathways through which they act. Such knowledge could provide valuable insights into their potential therapeutic applications.

Some of the effects observed in RtAE and its fractions have been previously studied in other plants of the genus *Rhus*, as have the underlying mechanisms, including the inhibition of NF-κB and the MAPK signaling pathway [52,60], the decrease in MCP-5 and Pro-MMP-9 cytokines [60], and the decrease in the JAK2/STAT3 pathway [61], among others. However, studies are needed to identify the main compound and its relationship with the underlying mechanism involved.

The LPS-induced rat paw edema model is a well-known model sensitive to COX inhibitors and has been used to evaluate the effect of nonsteroidal anti-inflammatory agents [62]. This work demonstrated the significant inhibition of COX-2 and IL-1β by F6. Consistent with the results observed in LPS-stimulated macrophages, protein expression in paw tissue was suppressed by F6. Therefore, F6 had a greater effect on gene expression than on COX-2 enzyme inhibition, as reported in previous studies, where the results suggest that the anti-inflammatory effects of F6 might be mediated by inhibiting the IL-1β and TNF-α genes [22].

However, COX-2 immunodetection did not decrease in response to RtAE or F3, likely because the compounds contained in those fractions, such as luteolin, epigallocatechin, apigenin, and quercetin, act by inhibiting COX-2 function and, consequently, PGE_2_ production.

The active site of COX-2 is known to consist of three regions: a hydrophobic pocket defined by Tyr385, Trp387, Phe518, Ala201, Tyr248, and Leu352; the entrance of the active site lined by the hydrophilic residues Arg120, Glu524, and Tyr355; and a side pocket lined by His90, Arg513, Val523, and Ser530 [63]. A docking study demonstrated that the catechol group of the B-ring of luteolin (a compound that RtAE also contains) was oriented toward the hydrophobic pocket, with 3′,4′-dihydroxy groups forming H-bonds with Tyr385 and Ser530, which blocked its activity. Additionally, the number of hydroxyl groups on the B-ring appears to be related to the molecular conformation that influences interactions between flavonoids and enzymes, such as tyrosine kinase and protein kinase C, involved in COX-2 transcriptional activity [64].

Treatment of mice with apigenin, which is also contained in RtAE, helped reduce skin tumor formation. In postmortem assays, the skin of the mice was analyzed by Western blot and ELISA, which revealed reduced levels of COX-2, PGE_2_, EP1, and EP2 (E-type prostaglandin receptors), as well as decreased cell proliferation [65].

These properties of reducing inflammatory mediators are similar to those of nonsteroidal anti-inflammatory drugs (NSAIDs), which have anti-inflammatory, antipyretic, and analgesic effects [66]. Therefore, the RtAE effect on LPS-induced acute inflammation might result from COX-2 inhibition, which consequently leads to the inhibition of prostaglandin synthesis and pro-inflammatory cytokine signaling.

The induction of COX-2 by Toll-like receptors activates the production of PGE_2_, which results in vasodilatation, chemotaxis, and the arrival of leukocytes, increasing the tumor volume; when COX-2 is inhibited, this effect does not occur; therefore, edema in the rats decrease. This effect is shown in Figure 4, where a decrease in cellular infiltration with Rt treatment was evident. Previous studies have shown that several aqueous extracts of various plants with high polyphenol contents act by exerting this mechanism of action [67,68].

## 4. Materials and Methods

### 4.1. Chemicals and Reagents

Prostaglandin E2 (Cat. 363246, Sigma-Aldrich©, St. Louis, MO, USA) was used (HPLC grade). The control drug was dexamethasone (DXM) (10 µM; D4902, Sigma^®^, St. Louis, MO, USA). The vehicle controls were 1× PBS (100 µL/day in animals) or 0.5% DMSO-1× PBS (*v*/*v* in cells; D2650, Sigma^®^, St. Louis, MO, USA). HPLC grade water, methanol, and acetonitrile were purchased from Tedia (Ohio, OH, USA). The additional use of equipment and reagents is indicated in the text.

### 4.2. Recollection of Plant Material

*Rhus trilobata* Nutt. (common name: skunkbush sumac; Family: *Anacardiaceae*; WFO ID:0001049775) was collected from Cerro Pelón, Municipality of Namiquipa (Chihuahua, Mexico) (INEGI topographic map H13C42 and geographical GPS coordinates: 29°5′59′′N, 107°32′33′′W to 1960 masl) in May 2015. The collected material was identified and validated according to the criteria described by Varela-Rodríguez et al. [18]. Leaves, fruits, and flowers were separated, and stems were cleaned to remove soil and insects. Stems were ground and sifted by a 0.5 mm sieve to be freeze-dried and refrigerated until used. 

### 4.3. Preparation of Plant Extracts and Fractionation

AERt was obtained by decoction (25 g of ground Rt stems were placed in an Erlenmeyer flask with 500 mL of distilled water and boiled for 30 min on a hot plate with constant stirring). At term, the decoction was filtered through Whatman #1 paper and centrifuged in 50 mL aliquots at 2500 rpm for 15 min at 4 °C. The recovered supernatant was named fraction 2 (F2), and an aliquot of 500 µL was reserved for characterization. Later, F2 (500 µL) was fractionated using ENVI™-C18 cartridges (Supelclean™, Sigma^®^, St. Louis, MO, USA) previously activated with absolute methanol (15 mL) followed by 1% acidified water (15 mL) in a vacuum manifold (Visiprep™, Sigma^®^, St. Louis, MO, USA). The compounds attached to the column were eluted using 1% acidified water (15 mL) (F3), ethylic ether (F4), ethyl acetate (F5), and methanol (F6). The fractions and AEs were concentrated under negative pressure with a rotary vacuum evaporator in a Büchi Rotavapor^®^ (R-300, Büchi Labortechnik AG, Flawil, CHE) and resuspended in phosphate-buffered saline (PBS) for in vitro and in vivo treatments. After the procedure, the AERt achieved a dry weight yield of 3.49 g/gRt. The dry weight yields of the fractions were F3 (610 mg/gRt), F4 (510 mg/gRt), F5 (400 mg/gRt), and F6 (0.3 mg/gRt).

### 4.4. Cell Culture

The murine macrophage line J774A.1 (MOs; TIB-67 ATCC^®^, Rockville, MD, USA) was generously provided by Dr. Patricia Talamás-Rohana (Centro de Investigación y de Estudios Avanzados, Mexico City, Mexico). The cells were seeded at a density of 3 × 10^6^ cells/mL in Dulbecco’s modified Eagle’s medium (DMEM) (Gibco™; Thermo Fisher Scientific, Inc., Waltham, MA, USA) supplemented with 10% (*v*/*v*) fetal bovine serum (FBS, Gibco™), 1% penicillin-streptomycin (10 mg/mL, Sigma^®^) and gentamycin (10 μg/mL, Sigma^®^). The cells were incubated at 37 °C with 5% CO_2_ (95% humidity) and harvested by Scraping.

### 4.5. Evaluation of the Cytotoxicity of Rhus trilobata to LPS-Induced MOs

The cytotoxic effects of RtAE and its fractions on cell viability were evaluated using MTT assays in 96-well plates, according to Montes-Fonseca et al. [69]. For this assay, 10^5^ cells were harvested in high-glucose DMEM. The cells were stimulated with 5 μg/mL LPS (type 0111: B4 from *Escherichia coli*; Sigma-Aldrich©, St. Louis, MO, USA); RtAE and fractions at 15 and 20 µg/mL were added to cultures and incubated for 24 h at 37 °C in a humidified atmosphere at 5% CO_2_. Cultures not LPS-stimulated were included as a negative control; cultures of LPS-stimulated MOs treated with 10 μM dexamethasone (DXM; Sigma^®^) or 5% dimethyl sulfoxide (DMSO; Sigma^®^) or left untreated were used as the anti-inflammatory positive control, death control, and anti-inflammatory negative control, respectively. After 20 h of incubation, MTT was added (0.5 mg/mL) to each well, and at term, the cells were lysed with acidified isopropanol, and absorbances at λ = 590 nm were obtained using a Varioskan^®^ Flash microplate reader (Thermo Scientific^®^, Inc. Waltham, MA, USA). Cell viability was calculated using the following formula: % viability = (absorbance of treatment/absorbance of negative control) × 100.

### 4.6. In Vitro Evaluation of the Anti-Inflammatory Activity of Rhus trilobata in LPS-Induced MOs

3 × 10^6^ J774A.1 MOs were seeded in a 6-well plate in supplemented high-glucose DMEM (Gibco™; Thermo Fisher Scientific, Inc., Waltham, MA, USA) and stimulated with 5 μg/mL LPS (type 0111: B4 from *Escherichia coli*; Sigma©). The cells were incubated with RtAE or its fractions at 15 µg/mL; cultures supplemented with DXM (10 µM) or left untreated were used as positive and negative anti-inflammatory controls, respectively. Cultures were incubated for 24 h, and at term, supernatants were collected and stored at −20 °C for further analysis. The MOs were detached, pelleted, and lysed for total RNA extraction.

Total RNA was isolated using TRIzol (Invitrogen, CA, USA) according to the manufacturer’s protocol and quantified and reverse transcription (RT)-quantitative polymerase chain reaction (qPCR) for IL-1β, IL-6, TNF-α, and COX-2 was performed using the primer sets listed in Table 1. The reference gene was glyceraldehyde-3-phosphate dehydrogenase (GAPDH) [70]. cDNA was synthesized from 1 µg of total RNA using the SensiFAST™ cDNA Synthesis Kit (Bioline, Memphis, TN, USA). For each gene, 3 µL of individual cDNA was subjected to qPCR with Thermo Scientific™ Maxima SYBR Green/ROX qPCR Master Mix (Thermo Scientific^®^, Inc., Waltham, MA, USA). qPCR was performed using a Quant Studio 3 PCR system (Thermo Scientific^®^ Inc., Waltham, MA, USA) with 1 cycle at 94 °C for 1 min, followed by 35 cycles of denaturation at 95 °C for 5 s and alignment/extension at 60 °C (RPL13a and IL-6), 56 °C (TNF-α), 50 °C (IL-1β), or 55 °C (COX-2) for 30 s. The sequences of the primers used are presented in Table 5. The real-time fluorescence data were collected during the elongation step of each cycle. Each cDNA sample was tested in duplicate. The relative quantification (RQ) was estimated with the ΔΔCt method (RQ = 2^−ΔΔCt^) [71]. The anti-inflammatory effect of RtAE and the fractions on inflammatory mediators’ RNA expression was analyzed by comparing the expression of treated or untreated LPS-stimulated MOs.

To determine the effect of RtAE and its fractions on PGE_2_ production, PGE_2_ was isolated from the culture supernatant using ENVI™-C18 cartridges (Supelclean™, Sigma^®^) and a method modified from Sánchez-Ramírez et al. [72]. Briefly, 0.5 mL of water-ethanol (1:4) and 10 µL of glacial acetic acid were added to 3.0 mL of each supernatant. After mixing well, the samples were incubated at room temperature for 3 min and then centrifuged at 12,000× *g* for 2 min. Each supernatant was loaded into an ENVI™-C18 cartridge previously activated with 20 mL of methanol, followed by 20 mL of acidified water (1% acetic acid). PGE_2_ was eluted with 2 × 0.75 mL of ethyl acetate; then, the samples were evaporated and reconstituted with a mobile phase solution (acetonitrile-methanol-deionized water 30:10:60) for high-performance liquid chromatography (HPLC) analysis.

**Table 5:** Primer sequences for quantitative PCR.

Gene	Primer Sequence
GAPDH [73]	F: 5′-TGAAGGTCGGTGTGAACGG-3´ R: 5′-GTGAGTGGAGTCATACTGGAA-3´
IL-1β [74]	F: 5′-GCAACTGTTCCTGAACTCAACT-3´ R: 5′-TCAACTGCCTGGGGTTTTCTA-3´
IL-6 [75]	F: 5′-TAGTCCTTCCTACCCCAATTTCC-3′ R: 5′-CTTCCTCACCGATTCCTGGTT-3′
TNF-α [76]	F: 5′-CCCTCACACTCAGATCATCTTCT-3´ R: 5´-GACATCGGGTGCAGCATCG-3′
COX-2 [69]	F: 5´- CTGTATCCCGCCCTGCTGGTG -3′ R: 5’-TTCTGTCGGTGGTAGTTGCGTTCA-3’

F, R; forward and reverse sequences.

HPLC analysis was performed with a Supelco Discovery C18 HPLC column (5 μm particle size, L × I.D. 15 cm × 4.6 mm). The HPLC instrument (Dionex, Sunnyvale, CA, USA) was equipped with a Dionex LPG-3400-D quaternary analytical pump, Dionex UltiMate 3000 diode array detector (DAD), Dionex solvent degasser, and Chromeleon CM-PCS-1 Software V. 6.80 SR12. An isocratic mode was used with a mobile phase of 0.01% acidified with a trifluoroacetic acid:acetonitrile:methanol (60:30:10) mixture. The UV detector wavelength was set to 210 nm, the flow rate was 0.8 mL/min, and the column and sampler rack compartment temperatures were 30 and 4 °C, respectively. The calibration curve was determined using the chromatographic peak areas corresponding to 0.625, 1.25, 2.5, 5, 10, and 20 mg/L PGE_2_ standard solutions using a mobile phase mixture as a standard dilution solvent. The samples were filtered through a 0.2 µm nylon syringe filter and injected into the HPLC instrument. The PGE_2_ concentration was determined through the peak area on the chromatograms.

### 4.7. Anti-Inflammatory Activity of Rhus trilobata in the LPS-Induced Paw Edema Model

Male Wistar rats aged 6–8 weeks and 250 g weight average (*n* = 42) were obtained from the Facultad de Ciencias Químicas *vivarium* from the Autonomous University of Chihuahua. The rats were housed at 24 ± 2 °C and 40 to 70% relative humidity under a 12 h dark/light cycle and were supplied with food and water ad libitum. This study was carried out following NOM-062-ZOO-1999 [Secretaría de Agricultura, Ganadería, Desarrollo Rural, Pesca y Alimentación, 2001] [77], International Guidelines (NC3Rs ARRIVE) and approved by the Institutional Animal Care and Use Committee (Authorization No. 012/16) from CICUAL-FCQUACH.

The animals were randomly divided into 8 groups of 3 rats each as follows: edema-positive control (C+), LPS-induced edema with no treatment; anti-inflammatory negative control (C−), LPS-induced edema treated with 50 μL of DXM (10 mg/mL); and six groups of LPS-induced edema treated with 500 µg of RtAE (50 μL of 10 mg/mL) and fractions F2, F3, F4, F5, or F6. In a second experiment, six additional groups of rats (3 in each) with LPS-induced edema were treated with 750 µg or 1000 µg of RtAE and fractions F3 or F6. At 0 h, the right hind paw of each rat was measured with a digital Vernier caliper (Truper^®^, Jilotepec, Edo. de México, Mexico), and 100 µg of LPS (type 0111: B4 from *Escherichia coli*; Sigma^®^) was immediately inoculated intradermally (i.d.) into the footpad. The treatments were applied 12 h after LPS induction. The anti-inflammatory effect (%) was determined by determining the increase in paw size at 12 h and 24 h, and % swelling was calculated using the following formula:

$$\%\;Swelling=\frac{\left[\left(12\;h-0\;h\right)-(12\;h-24\;h)\right]\;\times \;100}{Mean\;of\;swelling\;in\;C+}$$

The % anti-inflammatory effect was calculated by subtracting the % swelling of the different treatments from 100% (C+).

At 24 h, after the last measurement, the rats were euthanized with an overdose of sodium pentobarbital Sedalpharma^®^ (Pets Pharma, Edo. de Mex., Mexico), right hind paws were dissected and fixed in 4% buffered paraformaldehyde solution (Sigma^®^). Paraffin-embedded sections (4 μm thick) were prepared in an RM2125 RTS microtome (Leica, Buffalo Grove, IL, USA) and stained with hematoxylin and eosin (H&E; Merck, Darmstadt, Denmark) to assess histological damage using a BX41 Olympus microscope (Olympus Optical Co., Ltd., Miami, FL, USA) equipped with a Pixera-CCD camera (Santa Clara, CA, USA).

The effect of RtAE and its fractions on the expression of IL-1β and COX-2 was evaluated by immunohistochemical (IHC) analysis according to González-García et al. [49]. After blocking with 10% nonfat milk in PBS (pH 7.4), the slides were incubated separately for 1 h at 37 °C with a 1:100 dilution of rabbit anti-IL-1β or anti-COX-2 monoclonal antibodies (Santa Cruz Biotechnology, Dallas, TX, USA) in 1% nonfat milk in PBS. After two washes, the slides were incubated with a biotinylated anti-rabbit IgG secondary antibody for 1 h at room temperature. The signal was detected using avidin-peroxidase and diaminobenzidine substrate. The samples were counterstained with 1:10 hematoxylin and then dehydrated on permanent coverslips with Entellan resin. All the samples were processed on the same day to prevent variability. Controls with nonrelevant antiserum or without primary antibodies were used as nonspecific controls.

The expression of IL-1β and COX-2 was estimated by optical density (O.D.) as measured and analyzed with IMAGE Pro plus 4.1 software (Media Cybernetics, Silver Spring, MD, USA) [49]. Five representative microphotographs from each footpad at 60× were taken. After software calibration (individual pixel resolution of 175 gray levels), five measurements were made using a 50-pixel bar (*n* = 25). All the determinations were performed on the same day to reduce calibration or lighting errors. The samples used for image analysis were not counterstained to avoid background signals from hematoxylin.

### 4.8. Statistical Analysis

The data are presented as the mean ± standard deviation (SD). Where appropriate, comparisons between groups and controls were performed using ANOVA and Tukey’s test. Student’s *t*-tests were used to compare them with the reference control. Differences were considered significant when *p* ≤ 0.05. Statistical analysis was performed using Minitab software (State College, PA, USA).

### 4.9. Ultra-Performance Liquid Chromatography-Mass Spectrometry Analysis (RP-UPLC-PAD-QTOF-MS)

Compounds in F6 of RtAE were identified employing a UPLC Class I Acquity Waters^®^ (Milford, MA, USA) with photodiode array detector HL (PAD) coupled to high-resolution mass gasses Q-TOFMS (SynaptTM G1, Waters^®^). The instrument was equipped with a CSH C18 (2.1 mm × 150 mm, 1.7 µm, Waters^®^) UPLC column; the mobile phase used was acidified water (H_2_O with 0.1% formic acid, *v*/*v*; solvent A) and acetonitrile acidified (solvent B) (JT Baker^®^ (Phillipsburg, NJ, USA), MS grade). Compounds were eluted with a gradient separation as follows: 0 min, 5% B; 0.5 min, 5% B; 20 min, 75% B; 25 min, 75% B; 25.5 min, 90% B and held for two minutes for column washing; 27.6 min, 5% B and hold for 4.4 min for column re-equilibration. The sample was reconstituted in 2 mL of methanol (1 mg/mL), centrifuged, and filtered through 0.20 µm polytetrafluoroethylene (PTFE) syringe filter (Captiva Econo Filter, Agilent^®^ (Andover, MA, USA)), and maintained at 4 °C during the assay. A volume of 500 μL was diluted 1:2 with methanol and transferred to the vial for analysis. The chromatographic conditions were as follows: the flow rate was set at 0.2 mL/min throughout the gradient from the UPLC system into the MS detector. The injection volume was 10 µL, and the column temperature was maintained at 30 °C. The sample was analyzed by PAD in a wavelength range from 190 to 680 nm, and ions were generated by electrospray ionization source (ESI) using negative and positive modes. Spectra were acquired over a mass range from 50 to 1500 *m*/*z* using MSE acquisition mode. Precursor ion collision energy was set to 6 eV (trap section) and 20 to 40 eV in the transfer section. The optimum values of the ESI-MS parameters were capillary voltage, 2.5 kV for ESI- and 3.5 kV for ESI+; sampling cone, 35.0 V; extraction cone, 4.0 V; source and desolvation temperature, 150 °C and 350 °C; cone and desolvation gas flow, 20.0 L/h and 600 L/h, respectively. During acquisition, leucine enkephalin was used as a mass reference in 554.2615 for ESI- and 556.2771 for ESI+, which was infused directly at a flow of 5 µL/min at a concentration of 2 ng/mL, allowing internal mass calibration. MS data were acquired on continuum mode, exported to Progenesis^®^ QI for metabolites (Nonlinear Dynamics version 2.3, Waters^®^), calibrated in lock mass, and normalized. The maximum detection was from 0.5 to 28 min and adducts were programmed for automatic detection. The metabolites were putatively identified using Progenesis MetaScope for identification and HMDB (V.5.0), ChEBI, and Lipid Maps as search parameters; precursor tolerance of 20 ppm, theoretical fragmentation and fragment tolerance of 0 ppm, with a filter of isotope similarity of 90%. Afterward, a hand-curated database was conducted for each metabolite, and its putative candidates were identified using score punctuation ≥ 40 parameters and isotope similarity ≥90. The putative candidate with the higher parameters was the best. Once the metabolite had been identified, published information about it was sought to determine whether other researchers had reported it.

## 5. Conclusions

The results presented here demonstrated that RtAE and fractions F3 and F6 inhibited the inflammatory response by reducing the mRNA expression of IL-1β, IL-6, TNF-α, and COX-2 in LPS-induced MOs, as in a rat model of LPS-induced footpad edema.

Treatment with RtAE inhibited the inflammatory response by reducing the expression of IL-1β, TNF-α, and IL-6 in LPS-induced MOs in vitro and inhibiting acute LPS-induced inflammation in Wistar rats by suppressing COX-2 and IL-1β in inflamed tissue. We suggest that RtEA possesses significant anti-inflammatory activity and could be a potential source of naturally occurring anti-inflammatory agents.

## Figures and Tables

**Table 1:** Percentage decrease in PGE_2_ production in LPS-induced MOs by RtAE and fractions.

Sample	Concentration PGE_2_ (µg/mL)	PGE_2_ Decrease (%)
LPS-stimulated MOs (−)	6.30 ± 0.334 ^a^	
Nonstimulated MOs (+)	0.00 ± 0.085 ^f^	100
+DXM (10μM)	0.78 ± 0.198 ^e^	90.7
+AE (15 µg/mL)	2.87 ± 0.085 ^d^	53.68
+F2 (15 µg/mL)	2.64 ± 0.245 ^d^	54.72
+F3 (15 µg/mL)	0.68 ± 0.120 ^e^	88.91
+F4 (15 µg/mL)	4.43 ± 0.359 ^b^	24.02
+F5 (15 µg/mL)	3.60 ± 0.187 ^c^	45.78
+F6 (15 µg/mL)	0.55 ± 0.159 ^e^	90.12

LPS, lipopolysaccharide; (−), LPS-stimulated MOs without treatment; DXM, dexamethasone; AE, aqueous extract; F, fraction. Treatments that do not share the uppercase letter differ significantly (*p* ≤ 0.05), one-way ANOVA.

**Table 2:** Anti-inflammatory effects of RtAE and its fractions on LPS-induced paw edema in Wistar rats.

Treatment	Anti-Inflammatory Effect % (Range)	*p*-Value
DXM	93 (58 to 127)	0.005
AE	41 (19 to 62)	0.050
F2	23 (−20 to 66)	0.230
F3	59 (21 to 98)	0.028
F4	19.6 (−19 to 58)	0.250
F5	27 (−17 to 22)	0.200
F6	68.7 (10 to 127)	0.050

*n* = 3. Treatments with 500 µg of RtAE or the corresponding fraction. Differences were estimated using *t*-tests comparing each treated group with the non-treated group. A *p*-value of ≤0.05 was considered to indicate statistical significance. LPS, lipopolysaccharides; DXM, dexamethasone; AE, aqueous extract; F, fraction.

**Table 3:** Anti-inflammatory effects of different doses of RtAE, F3, and F6 on LPS-induced paw edema in Wistar rats.

Treatment	Concentration (μg)	Anti-Inflammatory Effects % (Range)	*p*-Value
DXM	500	93 (58.1 to 127.1)	0.005
AE	750	80 (9 to 151.8)	0.061
	1000	−27 (−85.1 to 30.7)	0.769
F3	750	36 (22.7 to 49.1)	0.005
	1000	37 (28.04 to 46.26)	0.001
F6	750	64 (17 to 111.8)	0.039
	1000	24 (−10.6 to 59.4)	0.147

*n* = 3. Differences were estimated using *t*-tests comparing each treated group with the non-treated group. A *p*-value of ≤0.05 was considered to indicate statistical significance. LPS, lipopolysaccharides; DXM, dexamethasone; AE, aqueous extract; F, fraction.

**Table 4:** Major metabolites of *Rhus trilobata* subfraction F6 by ESI(−) UPLC/MS-QTOF.

Peak No.	*m*/*z*	RT (min)	Maximum Abundance	Compound Name	Compound ID	DLMS	Biology Activity	Presence in Plants	Ref.
1	132.0554552	1.54	2.5	L-asparaginium	CHEBI:32651	−0.19	----	----	
2	275.9770172	8.65	2.6	Indole-3-carboxylic acid-O-sulphate	HMDB0060002	−1.49	Antiviral	----	
3	607.997306	9.22	7.1	9-ribosyl-trans-zeatin 5′-triphosphate(4-)	CHEBI:87953	0.38	Antioxidant, Neuroprotective	*Cocos nucifera*	[24,25]
4	652.0414331	9.4	5.3	ADP-L-glycero-D-manno-heptose(2-)	CHEBI:57564	0.82	Immunomodulator	----	[26]
5	260.200245	9.45	4.9	Butanamide	CHEBI:50724	−1.61	Anticancer, Anti-inflamatory, Neuroprotective, Antiviral	*Sedum ewersii Ledeb*	[27]
6	260.0158199	9.45	114.8	1-(2,2-Difluoroethyl) pyrrolidine-3,4-dicarboxylic acid	HMDB0257565	−0.14	Antitrombotic, Antidiabetic, Anti-inflammatory, Antineurogenic pain	----	[28]
7	517.8965765	9.97	7.4	UTP(3-)	CHEBI:57481	0.38	----	----	----
8	561.9356555	10.2	6.4	2-Fluoro-araatp	HMDB0245128	0.95	Anticancer, Antiviral, Immunomodulator, Antiinflamatory, Antioxidant, Cardioprotective	----	----
9	650.0239725	10.58	4.0	Adenophostin A	CHEBI:34524	0.99	Anticancer, Antiprotozoal, Antiviral, Imunomodulator	----	[29,30]
10	135.0054229	13.47	4.4	Tulipalin B	CHEBI:87123	−2.1	Anticancer, Antidiabetic, Antimicrobial, Antiinflamatory, Antiviral, Hepatoprotective, Immunomodulator	*Tulip gesneriana*, *Tulipa turkestanica*	[31,32]
11	224.0172865	13.73	7.4	2-(Methylthio)-3H-phenoxazin-3-one	HMDB0035996	−1.05	Antibiotic, Antiviral, Anticancer, Antioxidant, Anti-inflammatory, Neuroprotective,	----	[33]
12	205.0454102	13.78	4.9	Genipic acid	HMDB0036072	−0.75	Anticancer, Antimicrobial, Antiinflamatory	*Genipa americana*	[34]
13	348.003809	14.47	2.1	4-mercapto-6-oxo-3-phenyl-2-thiophen-2-yl-1,2-dihydropyrimidine-5-carbonitrile	CHEBI:105511	−0.65	Anticancer	----	----
14	242.1748489	16.02	2.4	N-undecanoylglycine	CHEBI:74438	−0.56	Antiviral, Antithrombotic, Immunomodulato, Neuroprotective	----	----
15	137.0262286	16.77	10.9	4-Methyl-3-oxoadipate-enol-lactone	CHEBI:81662	−1.55	Antiinflamatory, Antiviral, Antioxidant, Anticancer, Kidney protective	----	----
16	251.9897988	17.57	9.8	3-(acetamidomethylene)-2-(hydroxymethyl)succinate(2-)	CHEBI:19418	−0.82	Antiinflamatory, Antioxidant, Cardioprotective, Inmodulator, Neuroprotective	----	[35,36,37]
17	224.0155096	17.57	2.6	Kynurenic acid	HMDB0000715	−0.01	Cardioprotective, Kindney protective, Neuroprotective	*Taraxacum officinale*, *Urtica dioica*, *Chelidonium majus*, *Tripterygium wilfordii*	[38,39]
18	396.7970903	18.01	2.3	Iodic acid	CHEBI:24857	0.98	----	----	----
19	256.0360889	18.06	16.3	Unknown	----	----	----	----	----
20	262.9347679	18.42	6.3	D-xylono-1,4-lactone-5-phosphate(2-)	CHEBI:136751	−0.34	----	----	[40]
21	290.9780115	20.97	2.9	5-(3′-carboxy-3′-oxopropyl)-4,6-dihydroxypicolinate	CHEBI:2013	0.06	----	----	----
22	368.0782567	23.8	2.6	5′-O-beta-D-Glucosylpyridoxine	HMDB0246884	0.97	Anticancer, Cardioprotective, Neuroprotective	----	[41]
23	412.8722317	26.41	72.0	Arsonoacetic acid	CHEBI:28506	−0.63	----	----	----

Data show the results of the abundance of *m*/*z* values from three biological replicates. The putative compounds were identified based on the fragmentation pattern in concordance with ChEBI (https://www.ebi.ac.uk/chebi/init.do) (accesed on 25 September 2024), HMBD (https://hmdb.ca/) (accesed on 25 September 2024), and PlantCyc (https://www.plant.cyc.org/) (accessed on 22 September 2024) databases. The DMLS (drug-likeness model score) was calculated by Molsoft software (https://molsoft.com/mprop/) (accessed on 23 September 2024) with the parameters between −6 to −1 corresponding to non-drug compounds. The biological activity was clarified through the computational platform Way2Drug (https://www.way2drug.com/passonline/) (accessed on 25 September 2024) with a threshold of Pa = 0.5. Also, a scientific information search was conducted to find the compound that had already been reported. RT, retention time (min).

## Data Availability

All the data generated or analyzed during this study are included in this published article (as well as supplementary information files). The raw data are available upon request from the corresponding author.

## Supplementary Materials

The following supporting information can be downloaded at: https://www.mdpi.com/article/10.3390/plants13202840/s1, Text documents with fragmentation patterns of the F6 compounds.
