# Supplementary material for: Aqueous Extracts of Rhus trilobata Inhibit the Lipopolysaccharide-Induced Inflammatory Response In Vitro and In Vivo
Source: Plants (Basel). 2024 Oct 10;13(20):2840. doi: 10.3390/plants13202840 (PMC11514583; doi:10.3390/plants13202840)
Supplement: Supplementary file 1 [file plants-13-02840-s001.zip › plants-3209980-supplementary.pdf]

Fragmentation patterns of F6 putative compounds

Name: CHEBI:32651 (L-asparaginium)
ChEBI ID: CHEBI:32651
Precursor_type: [M-H]-
PrecursorMZ: 132.0555
Comment: 1.54_132.0555m/z
Formula: C4H9N2O3+
Num Peaks: 17
59.4701 3.1138
62.4156 1.9319
69.5929 5.9111
72.4995 8.7325
76.6479 7.2831
84.6550 2.0081
90.7207 77.3152
90.8292 6.3236
103.9442 6.7453
106.8057 9.3585
112.8454 1.5886
118.8853 1.1503
122.8519 41.9164
122.9779 3.2667
123.0294 0.2296
124.8562 6.3919
128.9021 3.6424

Name: HMDB0035996 (2-(Methylthio)-3H-phenoxazin-3-one)
DATABASE_ID: HMDB0035996
Precursor_type: [M-H2O-H]-
PrecursorMZ: 224.0173
Comment: 13.73_224.0173m/z
Formula: C13H9NO2S
Num Peaks: 50
54.3662 2.3137
55.2607 7.8347
64.5008 3.5290
66.5428 4.5952
68.5842 1.9918
72.4998 21.4913
74.5238 4.1435
76.6482 23.8450
78.6854 12.7943
80.7210 5.6290
90.7976 33.0787
91.8098 2.2286
92.8288 8.2968
94.8583 4.0947
98.7673 4.5282
102.8853 6.4627
104.9127 21.6474
106.9070 4.3341
106.9384 5.8480
114.9490 16.5067

116.9734 8.9872
118.9972 11.4634
120.9861 3.2107
121.0203 2.7840
122.0169 4.8823
122.9767 4.9115
128.0063 17.4876
131.0362 7.9601
133.0563 6.7821
135.0069 2.9966
137.0150 3.7893
139.0119 2.1007
141.0450 11.2944
145.0783 7.1584
147.0276 4.0342
147.0945 2.5308
149.0479 2.0862
151.0320 4.1172
153.0133 5.7907
157.0905 3.3153
159.1085 5.1196
161.0884 2.7338
163.0564 2.8827
165.0573 3.9218
167.0668 8.8655
177.0581 2.1774
181.0441 3.9916
191.0507 2.2830
207.0221 2.3303
224.0175 5.0646

Name: HMDB0036072 (Genipic acid)
DATABASE_ID: HMDB0036072
Precursor_type: [M+Na-2H]-
PrecursorMZ: 205.0454
Comment: 13.78_205.0454m/z
Formula: C9H12O4
Num Peaks: 50
55.2607 7.6462
64.5007 3.8340
66.5427 4.3500
72.4999 20.7713
74.5232 4.4133
76.6481 21.4526
78.6853 11.9424
80.7211 4.9677
90.7976 28.8854
91.8093 3.1510
92.8281 6.7592
94.8580 3.3598
98.7670 5.1033
102.8851 5.7700
104.9127 19.4162
106.9072 6.5168

106.9385 5.8794
108.9340 3.0675
114.9497 11.5563
116.9734 7.6981
118.9969 10.9460
120.9858 2.8865
121.0204 2.9425
122.0171 5.5405
122.9754 4.8659
126.9943 3.1496
128.0051 18.4490
131.0356 6.6278
133.0565 5.7213
135.0096 3.0244
135.0506 2.7427
136.9938 6.7313
141.0448 9.6814
145.0755 5.4053
147.0329 3.8591
147.0915 2.5145
151.0307 3.7919
153.0135 7.5424
155.0560 3.0914
157.0904 3.0449
159.1079 4.3307
161.0777 2.2823
165.0462 2.8454
167.0664 8.8018
173.0619 2.2739
177.0524 2.4239
181.0453 3.9332
183.0418 2.9036
191.0462 2.2650
201.0227 3.9359

Name: CHEBI:105511 (4-mercapto-6-oxo-3-phenyl-2-thiophen-2-yl-1,2-dihydropyrimidine-5-carbonitrile)
ChEBI ID: CHEBI:105511
Precursor_type: [M+Cl]-
PrecursorMZ: 348.0038
Comment: 14.47_348.0038m/z
Formula: C15H11N3OS2
Num Peaks: 50
54.3653 1.5811
55.2607 5.1275
68.5836 1.9663
71.4805 1.5112
72.5002 20.3610
74.5238 5.4576
76.6477 17.6824
78.6853 8.5590
80.7200 4.4837
90.7973 28.2019
91.8091 2.1563

92.8287 5.4745
94.8571 3.4031
98.7660 6.8129
102.8848 3.1220
104.9123 18.8035
106.9354 4.4892
114.9495 9.0211
116.9741 5.8909
118.9966 9.8273
120.9941 4.4157
122.0189 8.7185
124.9590 1.5713
126.9943 1.9902
128.0069 14.4904
131.0362 7.7655
133.0580 5.0614
135.0552 2.5773
139.0136 1.7352
141.0461 9.2802
145.0797 8.4933
151.0317 2.9985
155.0745 2.5496
157.0900 2.8255
159.1087 4.4494
161.0864 1.7936
163.0404 1.8106
165.0672 2.4160
169.0901 1.3407
171.1017 1.4711
173.1258 1.4640
177.0535 1.6921
179.0552 1.3462
187.0716 1.2361
191.0533 1.6153
201.1298 2.5433
230.1651 1.6456
230.9985 1.4738
248.9903 1.9194
286.8553 1.8850

Name: CHEBI:81662 (4-Methyl-3-oxoadipate-enol-lactone)
ChEBI ID: CHEBI:81662
Precursor_type: [M-H2O-H]-
PrecursorMZ: 137.0262
Comment: 16.77_137.0262m/z
Formula: C7H8O4
Num Peaks: 42
54.3656 1.7826
55.2608 4.6477
57.2945 1.2318
64.5003 4.4656
66.5435 3.9954
68.5842 1.9738
72.4998 19.8182

74.5235 6.5965
76.6475 18.3858
77.6628 0.1622
78.6851 12.3326
79.6929 0.2159
80.7210 7.7221
81.7265 0.0832
82.7387 0.2536
90.7978 25.5560
90.9078 0.4176
91.8094 1.4137
92.8280 5.6242
93.8346 0.3296
94.8575 3.0910
98.7664 7.7216
102.8854 2.0615
106.9348 5.2856
108.9326 2.9867
108.9661 1.5350
114.9488 7.0724
116.9742 4.7061
118.9981 8.8994
120.9568 0.5547
120.9932 2.1332
121.0181 2.5144
122.0194 11.4366
122.9732 1.0033
126.9930 0.9482
128.0065 11.4423
131.0373 4.9796
132.0380 0.3974
133.0572 5.4995
135.0627 6.0459
136.0611 0.2759
137.0248 9.8750

Name: CHEBI:136751 (D-xylono-1,4-lactone-5-phosphate(2-))
ChEBI ID: CHEBI:136751
Precursor_type: [M+K-2H]-
PrecursorMZ: 262.9348
Comment: 18.42_262.9348m/z
Formula: C5H7O8P-2
Num Peaks: 50
54.3661 1.5277
57.2926 1.8365
64.5002 11.8842
66.5424 4.0797
72.4996 18.5161
76.6478 22.8823
78.6854 10.8282
80.7221 4.6584
82.7239 2.3160
87.7953 0.5067
91.8088 0.8979

92.7946 4.5213
92.8244 4.8263
94.8586 3.0958
98.7670 8.9321
100.7747 0.5901
102.8852 1.3091
104.9127 15.1406
106.9362 5.5414
110.9626 0.7166
114.9508 3.4474
116.9744 2.8901
118.9976 7.3894
120.9482 9.7424
121.0135 2.8261
122.0197 13.7501
122.9759 0.9966
128.0065 8.1841
129.0172 5.0844
133.0592 3.5263
135.0659 9.2729
139.0125 0.6264
141.0456 1.6765
143.0647 2.5672
145.0811 10.5936
149.0092 24.3727
150.1147 9.2404
153.0161 1.2536
155.0769 1.0256
159.1083 2.3307
165.0581 0.8842
169.0916 0.6606
171.1069 1.3554
175.0091 15.0430
183.1020 0.8069
193.0265 8.6299
201.1254 0.5860
230.9943 1.0176
256.1306 4.2914
262.9343 0.6013

Name: CHEBI:2013 (5-(3'-carboxy-3'-oxopropyl)-4,6-dihydroxypicolinate)
ChEBI ID: CHEBI:2013
Precursor_type: [M+K-2H]-
PrecursorMZ: 290.9780
Comment: 20.97_290.9780m/z
Formula: C10H8NO7-
Num Peaks: 43
55.2613 1.9534
68.5828 0.8151
69.5930 0.8792
72.5001 15.7961
93.8340 0.3299
98.7690 8.7235
102.8832 0.4060

104.9118 10.0694
107.9396 1.4782
109.9466 0.2987
111.9696 0.1574
114.9493 1.4540
118.9977 4.9651
124.9366 0.2915
128.8221 0.7520
129.0164 1.4049
132.0380 0.0685
133.0537 2.7675
141.0441 0.5617
142.0538 0.5961
145.0797 2.2792
146.0665 4.7741
147.0776 5.1884
148.0847 0.1548
151.0405 0.2297
153.0151 0.8736
155.0771 0.6675
157.0936 1.0936
160.1057 0.1264
161.1021 6.9848
167.0769 0.2015
169.0950 0.3875
171.1065 0.5823
175.1099 1.5132
179.0836 0.0748
181.0789 0.1249
195.0795 0.1125
197.0979 0.0976
201.1281 0.2328
213.0822 0.0847
249.0577 6.3789
277.0314 0.7060
280.8617 0.2420

Name: HMDB0246884 (5'-O-beta-D-Glucosylpyridoxine)
DATABASE_ID: HMDB0246884
Precursor_type: [M+K-2H]-
PrecursorMZ: 368.0783
Comment: 23.80_368.0783m/z
Formula: C14H21NO8
Num Peaks: 39
54.3650 0.2718
57.2916 0.5625
66.5429 2.8628
69.5946 0.6108
72.4997 9.8475
74.5238 2.9987
76.6478 13.9912
78.6852 9.5128
81.7257 0.1550
88.7861 2.3797

94.8548 3.0541
95.8572 0.1291
98.7668 8.9062
104.9119 7.7943
106.9310 4.9439
108.9551 2.3612
110.9650 1.5526
118.9958 4.1203
120.9461 5.3485
121.0095 4.6794
122.0197 16.9825
128.0073 0.2492
131.0334 1.1320
134.0555 7.1015
135.0668 11.2536
142.0518 0.1408
143.0627 0.3336
146.0666 5.7579
147.0769 4.7626
149.0118 7.7225
150.1150 12.9007
153.0094 0.4785
157.0951 0.3801
161.1005 6.6932
164.1387 6.3158
173.1202 0.2915
314.5385 0.2600
318.5305 0.5402
368.0781 11.0015

Name: HMDB0060002 (Indole-3-carboxilic acid-O-sulphate)
DATABASE_ID: HMDB0060002
Precursor_type: [M+Cl]-
PrecursorMZ: 275.9770
Comment: 8.65_275.9770m/z
Formula: C9H7NO5S
Num Peaks: 50
55.2610 8.5521
64.5005 4.7758
66.5423 2.5478
69.5921 3.0451
72.5000 18.9827
76.6482 22.2646
78.6491 6.3749
78.6846 5.7042
80.6864 2.6982
84.7189 2.4832
90.7975 22.4568
92.7956 3.0115
92.8262 3.4527
94.8274 3.2420
96.8185 3.3696
102.8837 6.7858
104.9113 10.7880

106.9070 4.2748
110.9199 2.6942
114.9492 8.8501
116.9688 2.8015
118.9652 5.4952
118.9951 2.5647
122.9730 14.8268
124.9582 11.3381
126.9835 13.5269
131.0065 3.1779
133.0280 2.3992
135.0081 3.0227
137.0230 6.7482
139.0113 15.1510
145.0480 2.5708
147.0300 7.2792
151.0314 3.5981
153.0094 19.3285
154.0149 2.3086
157.0586 2.1992
161.0555 4.8047
163.0396 4.5746
165.0550 3.1998
167.0660 4.3276
179.0301 2.1095
181.0495 3.9379
189.0519 2.1296
193.0305 5.1105
206.9974 3.0063
216.9212 3.6192
228.9706 2.6652
240.9501 2.1986
270.8948 7.0219

Name: CHEBI:87953 (9-ribosyl-trans-zeatin 5'-triphosphate(4-))
ChEBI ID: CHEBI:87953
Precursor_type: [M+Na-2H]-
PrecursorMZ: 607.9973
Comment: 9.22_607.9973m/z
Formula: C15H20N5O14P3-4
Num Peaks: 50
55.2605 9.5536
64.5003 4.3719
69.5916 4.2432
72.4998 20.0023
76.6477 22.0188
78.6498 17.3539
80.6856 6.1385
90.7979 23.2265
92.7954 7.7759
96.8189 3.8380
102.8841 6.7135
104.9118 20.4276
105.9152 4.5078

106.9059 5.9439
108.8977 3.6708
114.9486 15.8031
118.9916 4.9190
120.9841 3.4526
122.9722 11.3938
124.9582 15.4990
126.9852 3.5266
131.0029 4.8135
132.0005 3.9154
133.0376 6.2417
135.0260 5.8915
136.9918 9.0433
139.0110 6.0684
141.0436 3.8469
145.0536 4.3527
147.0318 5.1066
151.0327 5.2688
153.0091 28.2776
160.0344 6.9735
161.0555 3.7743
163.0418 4.6688
167.0636 6.1027
216.9232 3.7941
228.9730 5.7216
230.9820 5.1520
240.9484 11.2215
258.9203 3.5492
270.8940 15.3173
280.8661 5.2770
282.8701 3.8329
286.8528 9.5994
302.8168 6.3796
314.8125 5.6569
318.7780 31.6717
392.7276 13.7498
422.7107 3.6137

Name: CHEBI:57564 (ADP-L-glycero-D-manno-heptose(2-))
ChEBI ID: CHEBI:57564
Precursor_type: [M+Cl]-
PrecursorMZ: 652.0414
Comment: 9.40_652.0414m/z
Formula: C17H25N5O16P2-2
Num Peaks: 50
55.2608 7.9022
64.5006 4.0355
69.5923 4.6466
71.6323 4.8397
72.5002 19.7438
73.6343 6.2522
76.6478 22.3683
78.6496 14.2757
80.6876 5.3976

90.7974 19.5461
97.8925 5.3711
102.8839 6.3444
104.9117 11.8241
106.9038 5.8944
108.8965 5.2202
114.9487 24.1755
116.0048 26.9720
118.9691 6.1856
122.9728 8.4966
124.9586 10.3917
126.9899 8.7264
128.0048 54.3389
129.0151 31.9585
133.0310 4.6245
135.0094 3.4279
136.9939 12.4119
139.0109 4.8589
141.0426 8.9491
145.0488 5.3843
147.0314 4.7529
151.0337 3.9538
152.0495 4.3999
153.0115 54.0280
155.0754 34.1070
157.0565 15.8270
161.0558 3.7756
163.0399 5.1552
165.0611 20.0138
167.0659 11.4647
181.0512 4.6954
183.0667 34.8389
216.9946 4.7305
218.0538 5.2105
244.9335 5.5588
254.9302 4.9151
260.0168 59.9858
272.8788 5.1899
280.8670 4.8583
286.8534 11.0575
318.7793 45.8865

Name: HMDB0257565 (1-(2,2-Difluoroethyl)pyrrolidine-3,4-dicarboxylic acid)
DATABASE_ID: HMDB0257565
Precursor_type: [M+K-2H]-
PrecursorMZ: 260.0158
Comment: 9.45_260.0158m/z
Formula: C8H11F2NO4
Num Peaks: 50
55.2607 7.9811
55.3767 4.7122
68.5124 3.0198
68.5466 1.2790

69.5920 3.6844
71.6320 8.1656
73.6342 9.8390
80.6877 4.5759
80.7204 1.5724
84.7181 1.1767
85.7952 2.1803
93.8129 1.1535
97.8921 9.9602
98.7675 1.2888
103.8927 0.5446
108.8955 7.6938
114.9488 19.6335
116.0052 32.8465
117.9684 0.9103
119.9939 0.9247
126.9906 12.9811
128.0049 48.3007
129.0153 26.1482
131.0079 6.1365
138.0017 3.0953
140.0230 0.5415
141.0433 8.7614
145.0481 5.8655
151.0350 3.6778
152.0499 8.3799
153.0547 25.6252
155.0762 29.8338
157.0577 19.1713
160.0335 1.0790
165.0169 3.5548
165.0610 19.4674
168.0554 6.1713
171.0413 1.6994
179.0298 3.3475
183.0669 37.2908
203.0097 2.1960
206.0148 0.6902
206.9960 2.7000
214.0086 0.4524
216.9926 5.2745
218.0526 1.8976
242.0633 0.7533
243.9346 0.5133
244.9318 6.5271
260.0178 71.9641
